# Biomimetic Cobalt Complex Stabilized by Hydrogel on High-Edge-Density Graphite for ORR and HER in Quiescent Solutions

**DOI:** 10.1021/acs.langmuir.5c01683

**Published:** 2025-08-19

**Authors:** Fhysmélia F. Albuquerque, Rodrigo M. Iost, Gabriel C. Fonseca, Radhakrishnan Venkatkarthick, Jessica C. Pacheco, Rafael N. P. Colombo, Fabio H. B. Lima, Frank N. Crespilho

**Affiliations:** † São Carlos Institute of Chemistry, University of São Paulo (USP), 13560-970 São Carlos, Brazil; ‡ Goiano Federal Institute of Education, Science and Technology, Campus Rio Verde, Rio Verde, GO 75901-970, Brazil; § Department of Fundamental Chemistry, Institute of Chemistry, 28133 University of Sao Paulo, Av. Professor Lineu Prestes, 748-B4T, Butantã, Sao Paulo 05508-000, Brazil; ∥ Chemistry Department, Federal University of São Carlos (UFSCar), 13565-905 São Carlos, Brazil; # BCMaterials, Basque Center for Materials, Applications and Nanostructures, UPV/EHU Science Park, Leioa, 48940 Leioa, Spain

## Abstract

Biomimetic catalysts are increasingly relevant in energy conversion due to their ability to imitate the redox activity and selective catalytic efficiency of natural enzymes involved in key reactions such as the oxygen reduction reaction (ORR) and hydrogen evolution reaction (HER). In this study, hydroxocobalamin acetate, a cobalt complex based on a corrin ring structure, analogue of vitamin B12 that mimics native active site of proteins, was immobilized on high-edge-density graphite electrodes (HEDGE). The exposed edge planes of HEDGE enhance electron transfer kinetics, providing a structurally favorable substrate for catalytic activity. The system was further encapsulated in an agarose hydrogel, which functions as a diffusion-regulating matrix, modulating gas and electrolyte permeability critical for sustained electrocatalytic performance in quiescent solutions. This configuration demonstrates dual catalytic functionality, efficiently facilitating ORR via selective molecular oxygen (O_2_) permeation and HER under mild conditions. Quiescent solutions, which mimic the diffusion-limited environments of natural enzymatic systems, present unique challenges such as gas bubble accumulation and restricted reactant transport. However, they also enable the investigation of intrinsic catalytic properties, offering biologically relevant insights into the system’s functionality. By emulating the microenvironmental conditions of natural enzymes, this hydrogel-based biomimetic system bridges the gap between biological principles and synthetic catalytic designs, providing a stable and efficient platform for electrocatalytic applications in energy conversion and storage technologies.

## Graphical abstract

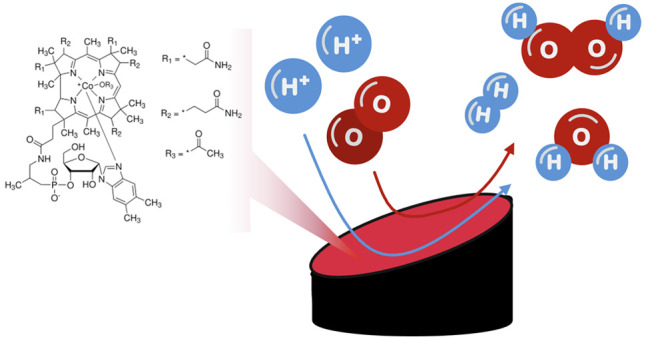

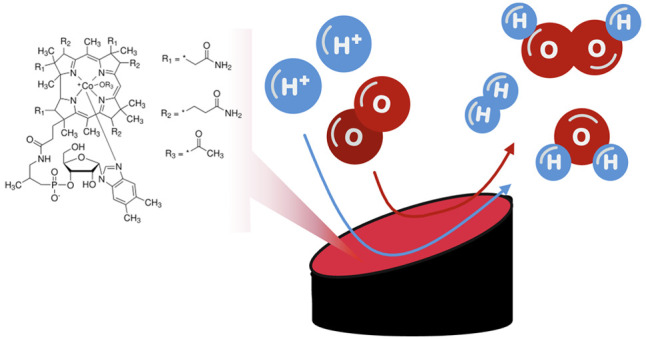

## Introduction

Cobalt macrocyclic complexes, such as cobalt porphyrins and corrin-based compounds, have gotten significant attention as biomimetic catalysts due to their structural and functional similarities to the active sites of natural metalloproteins.^1,2^ One such derivative, hydroxocobalamin acetate (CoP, a cobalt-based compound) closely mimics the prosthetic group of enzymes involved in biological processes such as oxygen reduction^3^ and hydrogen evolution.^4^ The coordination environment of cobalt in these compounds is key to their ability to catalyze reactions that are essential for energy conversion systems, positioning them as promising candidates for artificial enzyme development.^5^ The functional versatility of cobalt-based macrocyclic compounds, including porphyrins and corrin complexes, stems from their ability to engage in a wide range of redox processes, akin to the cobalt-containing cofactors found in natural metalloenzymes like nitrile hydratase or vitamin B12 and its derivatives.^6,7^ These prosthetic groups, present in enzymes responsible for critical biochemical reactions such as the oxygen reduction reaction (ORR) and hydrogen evolution reaction (HER), enhance catalytic efficiency and selectivity. Despite this potential, challenges such as limited long-term stability, inefficient immobilization on electrode surfaces, and restricted substrate diffusion remain key obstacles for their integration into practical catalytic systems. By emulating these biological systems, CoP offers an exciting avenue for developing artificial metalloenzymes tailored for sustainable energy applications.^8^

Quiescent solutions, characterized by the absence of forced convection or stirring, present challenges and opportunities in the study of electrocatalytic systems.^9^ The lack of bulk motion in these solutions often leads to diffusion-limited transport of reactants and products to and from the electrode surface, which can hinder reaction rates and mask intrinsic catalytic performance. Furthermore, the absence of stirring can exacerbate issues such as gas bubble accumulation at the electrode interface, uneven ion distribution, and local pH gradients, complicating the interpretation of experimental results.^10–12^ However, quiescent conditions are of particular importance as they closely mimic the environments in which natural enzymes operate. In biological systems, enzymes function in microenvironments where diffusion governs the availability of substrates and the removal of products, and forced convection is absent.^13,14^ These natural settings rely on finely tuned molecular architectures and local diffusion pathways to achieve high catalytic efficiency. By studying electrocatalytic systems under quiescent conditions, researchers can replicate these biologically relevant environments, gaining insights into the intrinsic performance of biomimetic catalysts and designing materials optimized for real-world applications that emulate natural processes.

In this study, CoP was immobilized on HEDGE and encapsulated within an agarose hydrogel layer to create a stable, robust, and efficient platform for dual electrocatalysis.^15–18^ The agarose hydrogel (Sac), chosen for its biocompatibility, tunable porosity, and mechanical stability, mimics extracellular matrices found in nature, further enhancing the biomimetic approach.^19^ This feature is particularly advantageous for studying electrocatalysis in quiescent solutions, where gas permeation and electrochemical stability are critical factors. By combining the biomimetic properties of CoP with the tailored environment provided by the hydrogel, this system mimics natural enzymatic processes while offering enhanced durability and functionality in synthetic settings. Our approach lays the groundwork for applications in energy conversion technologies, emphasizing the importance of material design and controlled environments in catalysis. The HEDGE, with its high density of active sites, not only supports efficient electron transfer but also ensures stable immobilization of the CoP, addressing a major limitation in conventional electrode systems.^20^

## Materials and Methods

### Materials

All chemicals were of analytical grade and used as received without further purification. Agarose (Kasvi) served as the hydrogel matrix, while glutaraldehyde (Sigma-Aldrich, ≥25%) was employed as a cross-linking agent to stabilize the hydrogel structure. CoP­(Sigma–Aldrich, Figure S1) was utilized as the catalytic active component. Sodium sulfate (Na_2_SO_4_, Êxodo Científica, 99%) was used as the supporting electrolyte, with pH adjustments performed using sodium hydroxide (NaOH, Sigma–Aldrich, 98%) and sulfuric acid (H_2_SO_4_, Sigma–Aldrich, 98%). All solutions were prepared with deionized water with a resistivity of ≥18 MΩ cm, ensuring minimal contamination and high solution purity.

### Biomimetic Hydrogel-Stabilized CoP on HEDGE

The development of the biomimetic hydrogel-stabilized CoP system on a HEDGE was carried out through a six-step process, each designed to enhance electrochemical performance and mechanical stability. In Step 1, the agarose hydrogel precursor (1.5 mg mL^–1^ in water) was thermally activated by heating to 85 °C under constant magnetic stirring for 10 min, ensuring complete solubilization and forming a homogeneous colloidal matrix. Upon reaching a semiviscous state, Step 2 involved cooling to a critical gelation temperature of approximately 50 °C, where glutaraldehyde (final concentration: 1.25%) was introduced as a bifunctional cross-linking agent. This step facilitated covalent network formation within the hydrogel matrix, enhancing its mechanical robustness. Step 3 incorporated CoP into the partially cross-linked hydrogel at a final concentration of 1.5 mg mL^–1^. Homogenization was achieved through vortex mixing, ensuring nanoscale dispersion of the CoP molecules within the polymer matrix, effectively creating a conductive catalytic network optimized for redox activity. The electrode preparation followed in Step 4, where graphitic HEDGE rods (6.1 mm diameter) were mechanically polished at a 45° angle using a silicon carbide 413Q 220-grit abrasive paper (3M), this polishing procedure increases the exposure of edge planes in the graphitic structure, resulting in an electrode with a greater density of accessible edge sites and exposed oxidized functional groups than untreated graphite. Then, the electrode was followed by ultrasonic cleaning in deionized water for 5 min to remove particulates and surface contaminants, resulting in a high-activity substrate. This procedure was adapted to previous work.^21^ Step 5 involved depositing, by drop casting, 50 μL of the Sac/CoP composite onto the polished electrode surface. To ensure strong adhesion and uniform distribution, the coated electrodes were dried under vacuum at room temperature for 60 min, effectively immobilizing the catalytic material onto the electrode. Finally, in Step 6, electrochemical testing was performed in a three-electrode cell configuration using a μ-Autolab Type III potentiostat/galvanostat at 25 °C. The Sac/CoP-modified HEDGE served as the working electrode, with a saturated Ag/AgCl electrode as the reference and a platinum wire as the counter electrode. A 0.1 mol L^–1^ Na_2_SO_4_ solution, adjusted to pH 5, 7, or 9 using H_2_SO_4_ or NaOH, was used as the supporting electrolyte to evaluate pH-dependent catalytic performance. To control the gas-phase environment, the electrolyte was purged with N_2_ (1.5 mL min^–1^) for inert conditions or O_2_ for oxidative conditions for 20 min prior to measurement. Electrochemical characterization, including cyclic voltammetry (CV) and linear sweep voltammetry (LSV), was conducted over a potential range of +0.7 V to −1.5 V versus Ag/AgCl_sat_ at a scan rate of 10 mV s^–1^. System stability and reproducibility were validated by analyzing the third scan cycle.

### Characterizations and Complementary Analyses

Online electrochemical mass spectrometry (EC-MS) was employed to monitor gas evolution in real time during bioelectrochemical reactions, offering information into reaction kinetics, product distribution, and catalytic mechanisms. Electrodes were prepared by depositing either Sac or Sac/CoP solutions onto Toray carbon paper (10% porosity) coated with a polytetrafluoroethylene (PTFE) layer (0.02 μm thickness; Gore-Tex). The coated electrodes were dried under vacuum for 60 min at room temperature to ensure uniform film adhesion and minimal residual solvents. The prepared electrodes were then mounted in polyether ether ketone (PEEK) holders designed for high chemical and thermal stability, equipped with additional PTFE gaskets to prevent electrolyte leakage. Electrical contact was established using titanium tape encased in Teflon to minimize corrosion and ensure reliable conductivity. The active surface area of the working electrode was precisely controlled at 0.38 cm^2^. These assemblies were integrated with a Pfeiffer Vacuum QMA 200 quadrupole mass spectrometer via a microchannel interface, enabling the real-time detection and quantification of gaseous products. Hydrogen evolution was monitored at *m*/*z* = 2, while other reaction products, such as oxygen (*m*/*z* = 32) and carbon dioxide (*m*/*z* = 44), were also analyzed to elucidate reaction pathways and efficiency under varying conditions.

Fourier-transform infrared (FTIR) spectroscopy was performed using a Bruker Tensor 27 spectrometer, covering the spectral range of 620–4000 cm^–1^ to probe functional groups in both Sac and Sac/CoP films. Spectra were analyzed to identify characteristic vibrational modes indicative of Sac cross-linking, CoP incorporation, and potential chemical interactions between the Sac matrix and CoP molecules. Raman spectroscopy complemented FTIR analysis by providing molecular-level information on vibrational and electronic structures. Dried films on glass slides were examined using a Horiba LabRam HR Evolution micro-Raman spectrometer equipped with a 633 nm excitation laser (20 mW power). Spectra were recorded over a broad range (50–3500 cm^–1^), enabling the identification of CoP electronic transitions, Sac network modifications, and any structural defects introduced during film preparation. Film morphology was analyzed using field-emission scanning electron microscopy (FE-SEM) on a JEOL, Model JSM-7200F microscope. Prior to imaging, samples were sputter-coated with a thin layer of gold to enhance conductivity and reduce charging effects. Imaging was conducted at an acceleration voltage of 5.0 kV, revealing the surface topography, Sac porosity, and CoP distribution within the films. High-magnification images provided insights into microstructural uniformity and potential aggregation phenomena that could influence electrochemical performance. The integration of online electrochemical mass spectrometry (EC-MS) analysis with advanced spectroscopic and microscopic techniques provided a multifaceted characterization of the Sac/CoP system. Real-time gas evolution monitoring elucidated catalytic activity and reaction pathways, while FTIR and Raman spectroscopy confirmed the chemical stability and structural integrity of the films. Morphological studies with FE-SEM offered complementary information on film architecture, enabling the optimization of electrode design for enhanced bioelectrochemical performance. This holistic approach underscores the potential of hydrogel-stabilized CoP films in electrocatalytic applications.

## Results and Discussion

The electrode preparation process, summarized in Figure 1a, consists of a series of steps designed to maximize the exposure of edge planes on the graphite surface while effectively integrating CoP within a Sac matrix. The sequence highlights key stages, including surface preparation through abrasive polishing, modification with the Sac/CoP composite, and final vacuum drying to achieve a uniform and durable coating. Initially, the graphite electrode undergoes mechanical treatment through abrasion at a controlled angle to ensure uniform exposure of the edge planes. Following this, the electrode is subjected to ultrasonic cleaning for 5 min to remove residual particles and contaminants introduced during the mechanical abrasion process, providing a clean and reactive surface for further modification. Finally, the functionalization process begins with the preparation of Sac solutions containing agarose (1.5 mg mL^–1^) and glutaraldehyde (1.25%). Then, the CoP (1.5 mg mL^–1^) was added to the Sac. A 50 μL aliquot of the Sac and Sac/CoP solution were carefully deposited, by drop casting, on the surface of the pretreated electrodes, ensuring uniform distribution and anchoring of the catalytic sites to the exposed edge planes. This matrix plays a dual role by stabilizing the CoP molecules and maintaining permeability for gas and electrolyte diffusion.^19^ The electrode is then allowed to dry under ambient conditions, resulting in the formation of a cohesive and stable Sac layer encapsulating the CoP.

**Figure 1:**
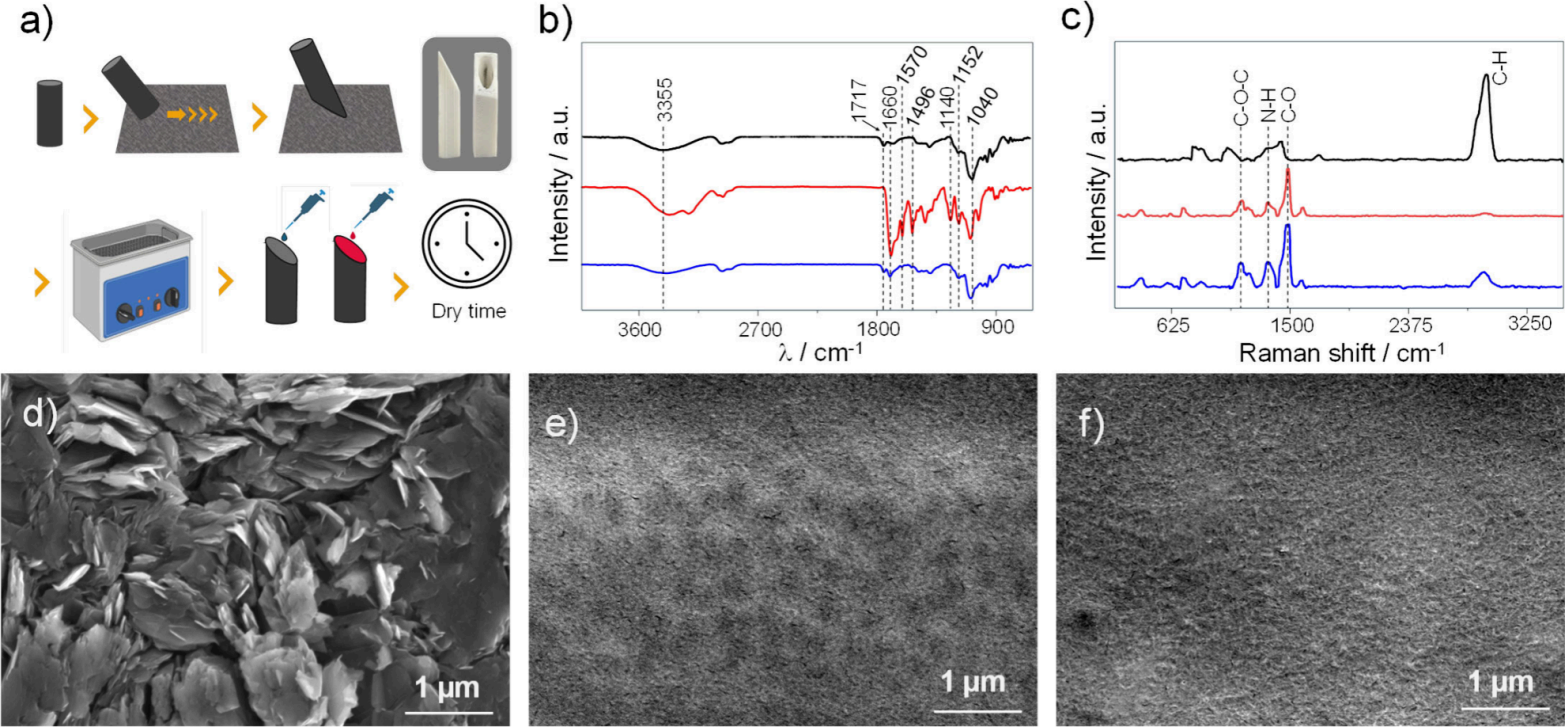
Electrode fabrication, spectroscopic characterization, and morphological analysis. (a) Schematic representation of the electrode preparation with a mechanical treatment to expose edge planes using a 3D mold, cleaning in an ultrasonic bath, and deposition of the material to be adsorbed. (b) FTIR spectra of analyzed in the range of 620–4000 cm^–1^ to identify the functional groups present in the material. (c) Raman spectra obtained in the range of 50–3500 cm^–1^ to investigate the vibrational features of the materials. (d–f) FE-SEM micrographs of HEDGE (panel (d)), Sac-coated HEDGE (panel (e)), and Sac/CoP (panel (f)). The micrographs highlight structural changes, from exposed graphite edge planes in panel (d) to a smooth Sac layer in panel (e) and a roughened surface in panel (f) due to CoP incorporation. The acceleration voltage used for SEM imaging was 5.0 kV. Scale bar = 1 μm.

The FTIR agarose spectrum (Figure 1b) exhibits a broad absorption band at 3355 cm^–1^, corresponding to O–H stretching broadened by hydrogen bonds, indicative of alcohol and hydroxyl groups.^22^ Peaks at 1152 cm^–1^ and 1040 cm^–1^ are associated with C–O stretching vibrations, characteristic of ether groups in the polysaccharide backbone. The Sac spectrum shows a broad O–H stretching band (2500–3300 cm^–1^) and a peak at 1717 cm^–1^, assigned to C=O stretching vibrations from cross-linking agents such as glutaraldehyde.^23^ Additional peaks at 1152 cm^–1^ and 1040 cm^–1^ align with those in the agarose spectrum, confirming its presence as the primary matrix material. The spectrum of CoP exhibits sharp peaks at 1660 cm^–1^ (C=O stretching of amides) and 1570 cm^–1^ (C=C stretching in the aromatic ring). The peak at 1496 cm^–1^ corresponds to C–N stretching vibrations,^24^ while the bands at 1140 cm^–1^ and 1057 cm^–1^ arise from C–O stretching of ester or ether groups associated with the CoP structure. The spectrum of the Sac/CoP system displays features from both the Sac and CoP, confirming the successful incorporation of CoP into the matrix. The broad O–H stretching band (2500–3300 cm^–1^) from the Sac and sharp peaks from CoP, such as the C=O band at 1660 cm^–1^ and the C=C band at 1570 cm^–1^, are present. Additionally, the C–N and C–O peaks from CoP (1496 cm^–1^ and 1140–1057 cm^–1^) are preserved, indicating the structural integrity of the CoP within the composite. The O–H and C–O peaks in the Sac spectra indicate the presence of a stable polysaccharide backbone. The C=O and C=N bands in the CoP spectrum confirm its corrin ring structure, and their presence in the Sac/CoP spectrum confirms effective incorporation. The combination of high wavenumber N–H stretch modes (3200–3400 cm^–1^) and aromatic (1550–1700 cm^–1^) peaks highlights the synergistic interaction between the Sac and the CoP.

In addition to conventional FTIR spectroscopy, which provides an average spectral response of the Sac/CoP system, micro-FTIR analysis was performed to investigate localized chemical interactions within the material. The micro-FTIR spectra (Figure S2) reveal spatial variations in functional group interactions, offering deeper insights into the structural modifications induced by CoP incorporation. These findings complement the global FTIR results, reinforcing the observed shifts in hydroxyl, carbonyl, and pyrrolic bands, which suggest strong molecular interactions between CoP and the Sac matrix. The hydroxyl stretching vibration, typically found in the 3200–3600 cm^–1^ range, exhibited a shift and intensity variation, indicating hydrogen bonding between the Sac network and phosphate or cobalt species present in CoP. Such interactions can modify the hydrogen bonding environment, leading to frequency shifts. Similarly, the carbonyl stretching mode, usually near 1720 cm^–1^, also shifted, suggesting potential interactions between the Sac’s carbonyl groups and CoP molecules. In related studies, the disappearance of the characteristic C=O peak and the emergence of new peaks corresponding to amide bonds were observed upon immobilization of cobalt phthalocyanine on graphene oxide, indicating strong interactions between the components.^25^ Collectively, these spectral shifts suggest that CoP not only integrates into the Sac but also establishes specific interactions at localized sites, altering the chemical microenvironment. These interactions are crucial as they can influence the material’s structural and functional properties, potentially enhancing its applicability across various fields.

The Sac Raman spectrum (Figure 1c) is dominated by characteristic peaks such as the (C–O–C) asymmetric stretching band at 1100 cm^–1^,^26^ indicating the polysaccharide backbone, and a strong C–H stretching band at 2900–3000 cm^–1^, representing aliphatic vibrations. These features confirm the presence of a stable and amorphous agarose matrix, essential for encapsulating catalytic species. In contrast, the CoP spectrum exhibits sharp and distinct features, including the Amide III band near 1200–1300 cm^–1^,^27^ corresponding to CoP amide groups, and a strong (C=C) aromatic ring stretching band at 1550 cm^–1^, reflective of the conjugated aromatic core of the corrin ring.^28^ A weaker C–H stretching band near 3000 cm^–1^ further highlights the aromatic structure of the CoP. In the Sac/CoP spectrum, key features from both components are retained, indicating successful integration of CoP into the Sac matrix. The enhanced (C=C) aromatic ring stretching band at 1550 cm^–1^ and the persistence of the (C–O–C) stretching band confirm that the structural integrity of both the CoP and the Sac is preserved. Minor shifts in the Amide III and aromatic ring signals suggest weak interactions, such as hydrogen bonding,^29,30^ between CoP and the Sac, without significant chemical modifications.

Figures 1d–f present FE-SEM micrographs of the surface of HEDGE (Figure 1d), Sac (Figure 1e), and Sac/CoP (Figure 1f), alongside an energy-dispersive X-ray spectroscopy (EDS) (Figure S3). Spectrum and the associated elemental composition table. The micrographs and compositional data together provide detailed understandings into the structural features and chemical makeup of the materials. The surface of the HEDGE electrode displays well-defined layered structures, characteristic of exposed edge planes of graphite.^31^ For Sac-coated HEDGE electrode, the smooth, uniform morphology indicates that the Sac layer successfully covers the underlying HEDGE substrate. This uniform coating ensures the Sac’s role as a stabilizing matrix for the subsequent incorporation of CoP. The Sac/CoP system appears slightly roughened compared to the Sac alone, likely due to the integration of CoP molecules. This confirms the structural compatibility of CoP with the Sac matrix. The EDS spectrum provides complementary compositional information, confirming the presence of cobalt from the incorporated CoP.^32^ The elemental composition table indicates the presence of carbon (49.82%), nitrogen (5.92%), and oxygen (40.31%), which align with the expected contributions from the Sac and CoP. The cobalt content (2.28%) confirms the successful incorporation of the CoP catalyst, while trace amounts of chlorine (1.67%) likely originate from precursor salts used in the synthesis or immobilization process.

The structural implications of the Sac/CoP electrode, as revealed by FTIR and Raman spectroscopy, feature the integration of CoP into the Sac matrix while maintaining the structural integrity of both components. FTIR analysis confirms the presence of functional groups characteristic of Sac and CoP, with overlapping peaks indicating effective incorporation. The Sac’s O–H and C–O signals establish the polysaccharide backbone as a stable matrix, while the retained C=O, C=C, and C–N features from CoP validate the structural integrity of the corrin ring. Additionally, the Raman data corroborate these findings, showing persistent peaks for the (C=C) aromatic ring and Amide III vibrations of CoP alongside the hydrogel’s polysaccharide signals. Minor spectral shifts suggest weak interactions, such as hydrogen bonding, between the components, which do not compromise the individual functionalities. The MEV-FEG micrographs reveal the physical coverage and distribution of the Sac and CoP on the HEDGE electrode. The uniform morphology of the Sac confirms its role as a stabilizing matrix, as also supported by the FTIR data. The FTIR spectrum of the Sac shows characteristic O–H stretching vibrations and C–O stretching, which are preserved in the Sac/CoP system. These spectroscopic features validate that the Sac matrix remains chemically intact during the incorporation of CoP, as corroborated by the smooth morphology observed in the micrograph. The Sac/CoP exhibits a slightly roughened texture, compared to the Sac alone, indicative of the integration of CoP molecules. This structural feature aligns with the Raman and FTIR data, which show the retention of CoP’s characteristic aromatic C=C stretching and Amide III bands, along with the Sac’s polysaccharide backbone signals. The Raman spectrum further highlights minor shifts in these bands, suggesting weak interactions, such as hydrogen bonding, between the Sac and CoP, which likely contribute to the uniform distribution and stability of the CoP within the matrix. The EDS spectrum and elemental composition confirm the presence of cobalt (2.28%) in the Sac/CoP system, directly linking the morphological observations to the successful incorporation of the catalytic CoP molecules. Additionally, the nitrogen content and the consistent presence of oxygen reinforce the structural contributions of both the CoP and the Sac. These results are consistent with the spectroscopic evidence, which identifies intact functional groups (C=O, C=C, and N–H) from both components, confirming that the CoP retains its catalytic functionality within the Sac matrix. Together, the morphological data confirm the physical integration and uniform distribution of the Sac and CoP, while the spectroscopic analyses validate their chemical stability and functional compatibility.

### ORR and HER

In contrast to glassy carbon, HEDGE electrodes is a promisor material for the immobilization of organic molecules due to its high porosity, which provides a large surface area for molecular attachment. The high density of carbon edge sites, which are highly reactive and facilitate strong interactions with functional groups, favors the adsorption of Sac/CoP. Additionally, the material’s structural stability and conductivity ensure efficient heterogeneous electron transfer reaction, making it ideal for applications in HER systems (Figures S4a and S4b). In Figures 2a and 2b, recorded under a N_2_ atmosphere to evaluate the HER, the cyclic voltammograms reveal significant differences in cathodic current density between the Sac (dotted lines) and Sac/CoP (continuous lines) systems. At pH 5, the Sac/CoP system achieves a cathodic current density of approximately −1.2 mA cm^–2^ at −1.5 V (vs Ag/AgCl), indicative of enhanced HER kinetics due to the abundance of protons in the acidic environment. As the pH transitions to neutral (pH 7) and alkaline (pH 9) conditions, the cathodic current densities decrease, with values of −0.9 mA cm^–2^ and −0.6 mA cm^–2^, respectively, at the same potential. This decrease reflects the kinetic limitation imposed by reduced proton availability at higher pH. Conversely, the Sac system displays negligible HER activity across all pH values, as evidenced by significantly lower current densities, highlighting the indispensable role of CoP as the active catalytic site in facilitating proton reduction.^33^

**Figure 2:**
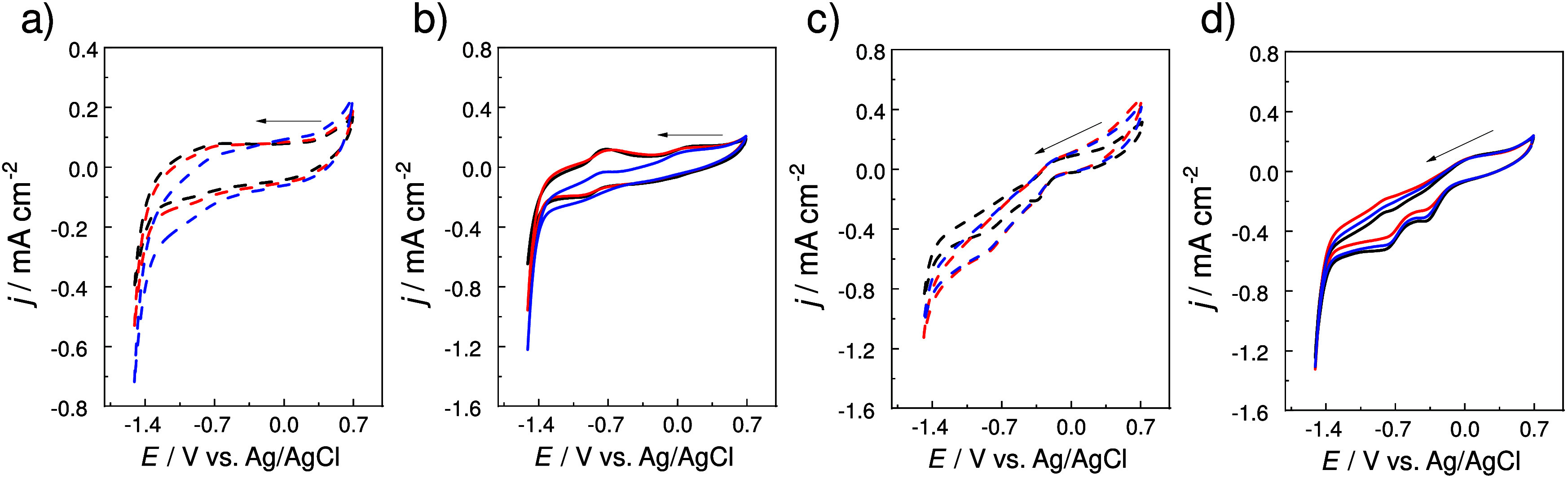
pH-dependent electrochemical response of Sac and Sac/CoP modified electrodes under inert and oxidizing atmospheres: cyclic voltammograms of HEDGE/Sac (dotted lines) under (a, b) N_2_-saturated solutions and (c, d) HEDGE/Sac-CoP (solid lines) O_2_-saturated at pH 9 (black), pH 7 (red) and pH 5 (blue). Supporting electrolyte: Na_2_SO_4_ 0.1 mol L^–1^. Scan rate = 10 mV s^–1^.

In Figures 2c and 2d, obtained under an O_2_ atmosphere to investigate the ORR, the Sac/CoP system demonstrates a catalytic activity compared to the ORR. At pH 9, the Sac/CoP system exhibits the highest ORR current density, reaching approximately −0.5 mA cm^–2^ at −0.8 V (vs Ag/AgCl). At pH 7 and 5, the current densities decline to −0.43 mA cm^–2^ and −0.48 mA cm^–2^, respectively, at the same potential. The Sac system, in contrast, displays negligible ORR activity across all conditions, emphasizing the essential role of CoP in mediating oxygen reduction.

Figure 3 compares cyclic voltammograms for the cathodic region of HEDGE electrodes modified with Sac (dotted lines) and Sac/CoP (continuous lines) under N_2_ (dark lines) and O_2_ (light lines) atmospheres at pH 9, pH 7, and pH 5. CoP shows mixed valence and two redox pairs were observed in the CVs. The oxidation redox peaks at −0.7 V and +0.2 V refer to the oxidation of Co­(I) → Co­(II) (*E*_p_ = −0.7 V) and Co­(II) → Co­(III) (*E*_p_ = +0.2 V). The reduction peaks at −0.8 V and −0.2 V refer to the reduction of Co­(III) → Co­(II) (*E*_p_ = −0.2 V) and Co­(II) → Co­(I) (*E*_p_ = −0.8 V).^34,35^ Under N_2_ conditions (Figure 3a, pH 9), the Sac/CoP system achieves a cathodic current density of approximately −0.6 mA cm^–2^ at −1.5 V, confirming CoP’s catalytic activity for HER at alkaline pH, despite limited proton availability. Under O_2_, ORR onset occurs around −0.3 V, with a maximum cathodic current density of approximately −0.5 mA cm^–2^. The Sac system shows negligible catalytic activity for both HER and ORR, reinforcing the significance of the CoP. In Figure 3b (pH 7), the Sac/CoP system exhibits enhanced HER performance under N_2_, achieving a current density of approximately −0.9 mA cm^–2^ at −1.5 V, indicating improved HER kinetics due to increased proton availability at neutral pH. Under O_2_, the ORR onset shifts to −0.4 V, with a maximum current density of approximately −0.4 mA cm^–2^, further confirming the pH-dependent catalytic activity of CoP. In Figure 3c (pH 5), the Sac/CoP system achieves its highest HER activity under N_2_, with a cathodic current density of approximately −1.2 mA cm^–2^ at −1.5 V. Under O_2_, the ORR current density decreases to approximately −0.3 mA cm^–2^, consistent with the higher overpotentials required for oxygen reduction under acidic conditions. Figure 3d presents quasi-steady-state linear sweep voltammograms for HER, with the onset potentials, for HER and ORR, presented in Table 1. At pH 5, the HER begins at −1.19 V vs Ag/AgCl, representing the least negative onset potential, which can be attributed to the higher proton availability under acidic conditions. As the pH increases to 7.0 and 9.0, the onset potential becomes more negative, reaching −1.26 V and −1.28 V, respectively. This trend reflects the increasing energetic requirement for HER at higher pH due to the reduced concentration of protons. In the context of HER, the thermodynamic potential shifts with pH due to proton availability, following eqs 1, 2, and 3:^36^

$$E_{H^{+}/H_{2},}={E^{{}^{\circ}}}_{H^{+}/H_{2}}-0.059\times \mathrm{pH}$$ 1

$$E_{H^{+}/H_{2},\mathrm{Ag}/\mathrm{AgCl}}=E_{H^{+}/H_{2}}-E_{\mathrm{Ag}/\mathrm{AgCl}}$$ 2

$$E_{H^{+}/H_{2},\mathrm{Ag}/\mathrm{AgCl}}=-E_{\mathrm{Ag}/\mathrm{AgCl}}-0.059\times \mathrm{pH}$$ 3

where *E*_Ag/AgCl_ is equal to +0.197 V vs SHE; at pH 5, the thermodynamic potential is approximately −0.492 V vs Ag/AgCl, while at pH 7 and pH 9, it shifts to −0.610 V and −0.728 V vs Ag/AgCl, respectively. Comparing these thermodynamic values to the observed onset potentials, the overpotentials for HER at −1.28 V (pH 9), −1.26 V (pH 7), and −1.19 V (pH 5) reveal an increasing trend in overpotential with increasing pH. This behavior reflects the greater kinetic barriers associated with the lower proton availability under alkaline conditions. However, the more negative thermodynamic potentials at higher pH levels indicate a higher energy requirement for HER, which is consistent with the observed trend of increasing overpotential.^37^ The catalytic proficiency of the Sac/CoP system was further assessed through turnover metrics. The estimated turnover number (TON) of 0.135 ± 0.018 and turnover frequency (TOF) of 0.135 ± 0.018 s^–1^ attest to the system’s capacity to sustain the HER over extended periods, with catalytic sites undergoing efficient substrate turnover.^38^ Notably, this TOF is significantly higher than that reported for a cobalt porphyrin catalyst in acidic media (pH 4.1) which exhibited a maximum TOF of 7.2 h^–1^ (equivalent to 0.002 s^–1^),^39^ reinforcing the high catalytic efficiency of the Sac/CoP system.

**Figure 3:**
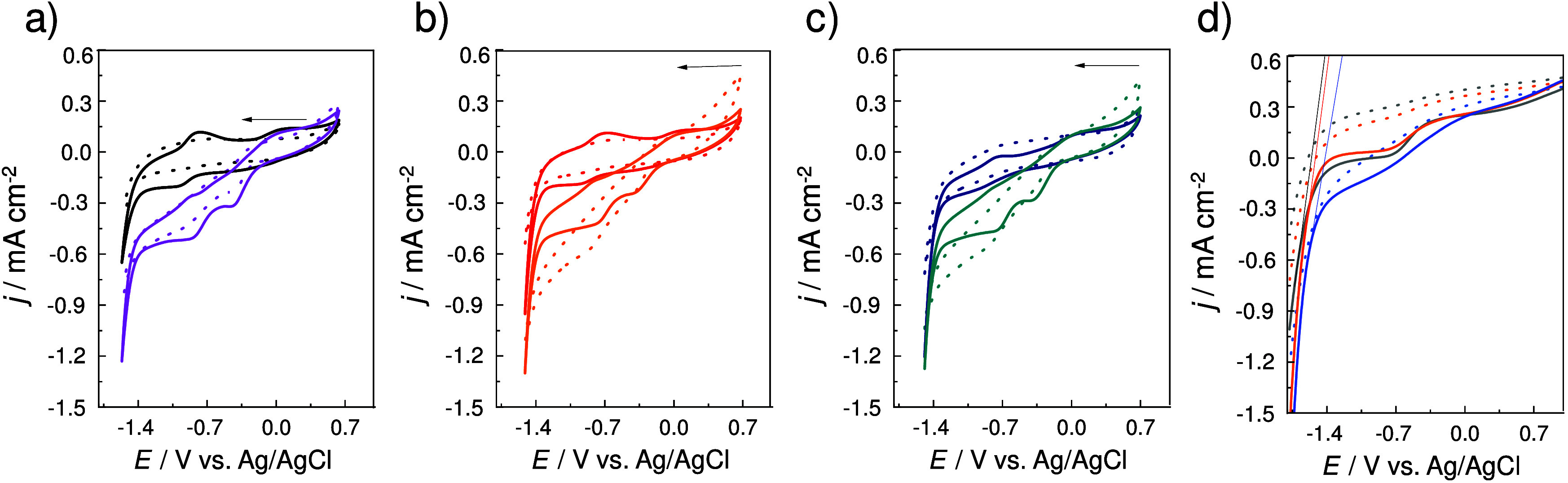
Influence of gaseous atmosphere on the electrochemical response of Sac and Sac/CoP electrodes: cyclic voltammograms of HEDGE/Sac (dotted lines) under N_2_-saturated solutions (a–d) and HEDGE/Sac/CoP (solid lines) O_2_-saturated (a–d) at pH 9 (panel (a)), pH 7 (panel (b)), and pH 5 (panel (c)). (d) Determination of onset potential under inert conditions: LSV for HEDGE/Sac (dotted lines) under N_2_-saturated solutions and HEDGE/Sac/CoP (solid lines) at pH 9, pH 7 and pH 5 under N_2_-saturated solution. Supporting electrolyte: Na_2_SO_4_ 0.1 mol L^–1^. Scan rate = 10 mV s^–1^.

**Table 1:** Comparison of Key Catalytic Parameters for Cobalt Porphyrin-Based Systems Reported for HER and ORR under Different pH Conditions^a^

reaction	catalyst	pH	*E*_onset_(*E*/V vs Ag/AgCl)	current density (mA cm^–2^)	turnover frequency, TOF (s^–1^)	turnover number, TON	ref
**HER**	Sac/CoP	5	–1.19	–1.2 at −1.5 V	0.135	0.135	This work
	Sac/CoP	7	–1.26	--	--	--	This work
	Sac/CoP	9	–1.28	--	--	--	This work
	Co-PB-1(8)^a^	7	--	--	0.22	19.03	^54^
	Co-TPP^b^	7	--	--	0.10	8.71	^54^
	Co-PPh_3_/G^c^	14	–1.47	166 at −1.9 V	--	--	^55^
	1-Co-py/G^d^	14	–1.47	100 at −1.9 V	--	--	^55^
	1-Co-py/G^e^	14	–1.47	73 at −1.9 V	--	--	^55^
**ORR**	Sac/CoP	5	–0.6	--	--	--	This work
	Sac/CoP	7	–0.4	--	--	--	This work
	Sac/CoP	9	–0.3	–0.5 at −0.8 V	--	--	This work
	CoTMPP^f^	14	–0.3	--	--	--	^56^

a Current densities are listed at the specified applied potentials.

b Cobalt porphyrin-based porous organic cage.

c Cobalt tetraphenylporphyrin;

d Co­(III) complex of 5,15-bis­(pentafluorophenyl)-10-(4-(1-pyrenyl)­phenyl)­corrole with axial triphenylphosphine ligand.

e Co­(III) complex of 5,15-bis­(pentafluorophenyl)-10-(4-(1-pyrenyl)­phenyl)­corrole with axial pyridine ligand.

f Co­(III) complex of 5,10,15-tris­(pentafluorophenyl)­corrole with axial pyridine ligand.

g Cobalt tetra-methoxy-phenyl porphyrin.

To contextualize the HER performance of Sac/CoP, key catalytic parameters were compared with cobalt-based systems previously reported in the literature (Table 1). Although some systems operating under strongly alkaline conditions (e.g., Co-PPh_3_/G or 1-Co-py/G) report high current densities (>70 mA cm^–2^), these results are often obtained at more negative potentials (−1.9 V vs Ag/AgCl). In contrast, Sac/CoP achieves a cathodic current density of –1.2 mA cm^–2^ at –1.5 V in quiescent solution at pH 5, with a TOF of 0.135 s^–1^. These values are comparable to the performance reported for molecular catalysts like Co-PB-1(8) (TOF = 0.22 s^–1^) and Co-TPP (TOF = 0.10 s^–1^). The combination of moderate overpotentials, measurable turnover metrics, and operation in unstirred media highlights the intrinsic catalytic efficiency of Sac/CoP and reinforces its relevance as a functional model for hydrogenase-mimetic systems.

For the ORR, the thermodynamic potential also varies with pH,^40^ following the eq 4, 5 and 6:

$$E_{O_{2}/H_{2}O}={E^{{}^{\circ}}}_{O_{2}/H_{2}O}-0.059\times \mathrm{pH}$$ 4

$$E_{O_{2}/H_{2}O,\mathrm{Ag}/\mathrm{AgCl}}=E_{O_{2}/H_{2}O}-E_{\mathrm{Ag}/\mathrm{AgCl}}$$ 5

$$E_{O_{2}/H_{2}O,\mathrm{Ag}/\mathrm{AgCl}}=E_{O_{2}/H_{2}O}^{{}^{\circ}}-E_{\mathrm{Ag}/\mathrm{AgCl}}-0.059\times \mathrm{pH}$$ 6

while $E_{O_{2}/H_{2}O}^{{}^{\circ}}$ is equal to +1.23 V vs SHE, the thermodynamic potential of ORR is approximately 0.738 V (pH 5), 0.620 V (pH 7), and 0.502 V (pH 9) all vs Ag/AgCl. The shift toward more negative onset potentials as pH increases reflects the higher energy barrier for ORR under alkaline conditions despite improved kinetics in acidic environments. As expected, the data confirm that pH significantly influences the overpotential for both HER and ORR, with acidic conditions favoring proton-driven HER and alkaline conditions slightly improving ORR performance relative to the thermodynamic baseline. CoP are versatile catalysts for HER and ORR due to their ability to mediate electron–proton transfers and stabilize reaction intermediates. In HER, CoP facilitates the reduction of H^+^ through a sequence of reaction steps: the cobalt ion center transitions from Co­(II) to Co­(I) binds a proton to form a cobalt-hydride (Co–H), and releases molecular hydrogen (H_2_). In ORR, CoP activates O_2_ by forming a Co–O_2_ reaction intermediate, which undergoes proton–electron transfers, either fully reducing O_2_ to H_2_O in a 4-electron pathway or forming H_2_O_2_ in a 2-electron reaction pathway.^35,41^ In Sac/CoP system, RRDE data (Figure S7) indicate that the reaction proceeds predominantly through the 2-electron pathway. This is supported by the ring current observed under oxygen-saturated conditions, consistent with the formation and detection of H_2_O_2_, as reported in established RRDE protocols.^42^

Figure 4 provides complementary evidence of HER activity via EC-MS and chronoamperometry.^43^ In Figure 4a, the ion current for H_2_ (*m*/*z* = 2) increases with more negative potentials, with pH 5 producing the highest ion current at −2.0 V, followed by pH 7 and pH 9. This trend aligns with the greater proton availability at lower pH. The chronoamperometric data in Figure 4b corroborates this trend, with stable cathodic current densities across all potential steps, further indicating the robustness of the Sac/CoP system. Figure 4c depicts Tafel plots, where the Tafel slope at pH 5 (266 mV dec^–1^) suggests a Volmer-type reaction as the rate-determining step (RDS), involving proton adsorption. At pH 7 and pH 9, the Tafel slopes increase to 333 mV dec^–1^ and 338 mV dec^–1^, respectively, indicating a transition to slower HER kinetics due to reduced proton availability.^44^

**Figure 4:**
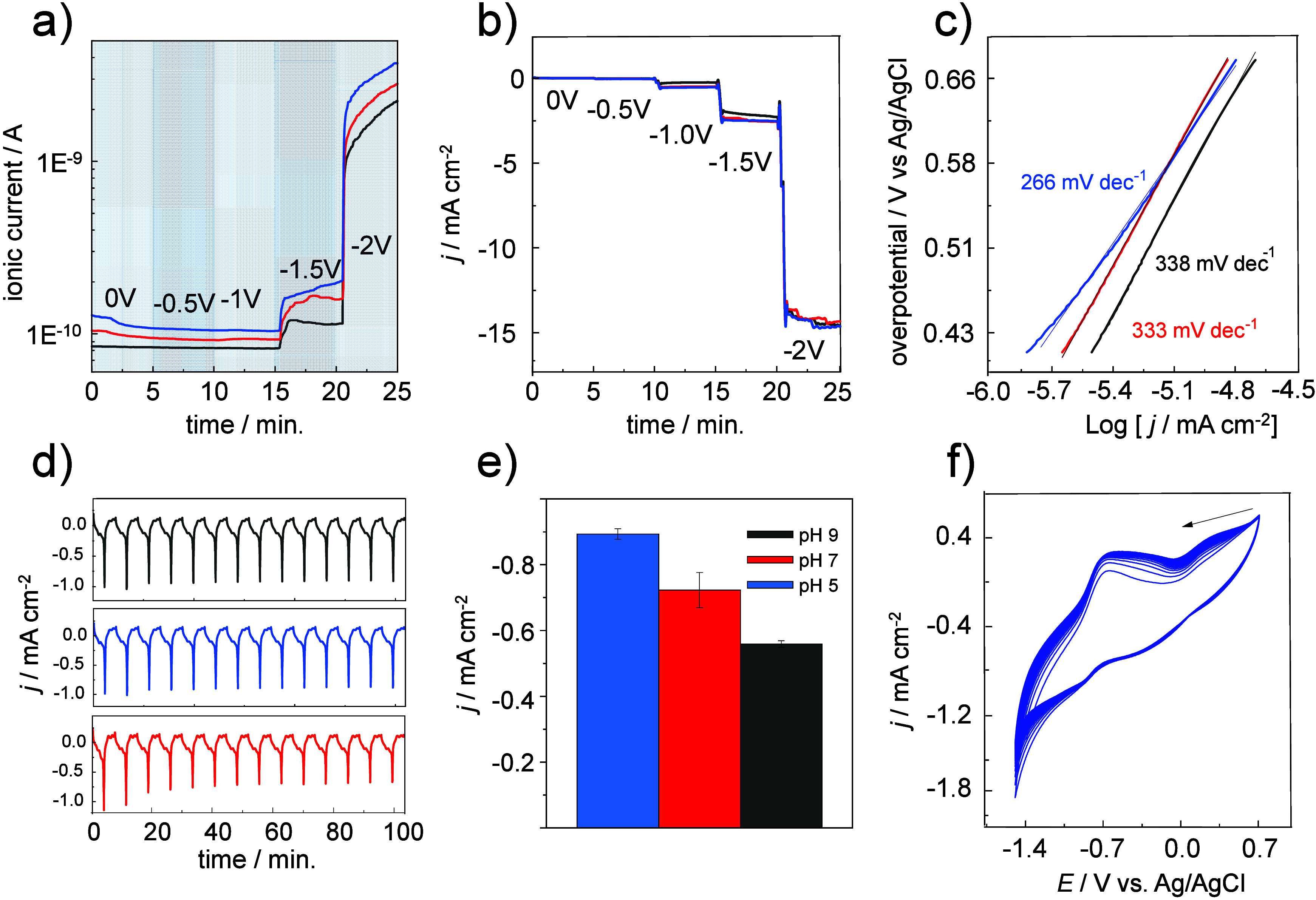
pH-dependent hydrogen evolution activity and electrochemical stability of the Sac/CoP electrode. (a) Differential electrochemical mass spectrometry (DEMS) for HER (*m*/*z* = 2), highlighting the effect of pH on HER activity. (b) Chronoamperometric profiles showing stable cathodic current densities for the HEDGE/Sac/CoP system across different pH conditions. (c) Tafel plots derived from LSV data, illustrating the HER kinetics at pH 5, pH 7, and pH 9. Supporting electrolyte: Na_2_SO_4_ 0.1 mol L^–1^. N_2_-sat conditions. (d) Long-term stability of current density for HEDGE/Sac/CoP, as a function of time up to 14 scans at pH 9 (green), pH 7 (red), and pH 5 (blue). (e) Scale bar for the change in current density at pH 9 (green), pH 7 (red), and pH 5 (blue). (f) Voltametric stability of HEDGE/Sac/CoP up to 50 cycles. N_2_-saturated solution. Supporting electrolyte: Na_2_SO_4_ 0.1 mol L^–1^. Scan rate = 10 mV s^–1^.

The results reveal a clear dependency on pH, the catalytic role of CoP, and the influence of the Sac matrix in maintaining electrode stability and facilitating gas and electrolyte diffusion. The HER performance of the Sac/CoP system is evident from the cyclic voltammograms and is further supported by the linear sweep voltammograms, Tafel plots, and EC-MS data. At acidic pH (pH 5), the Sac/CoP system exhibits the highest HER activity, achieving a cathodic current density of approximately −1.2 mA cm^–2^ at −1.5 V. The EC-MS analysis confirms that H_2_ generation is maximized at this pH, as indicated by the ion current at −2.0 V. The Tafel slope of 266 mV dec^–1^ suggests that the HER proceeds via a Volmer-type rate-determining step, consistent with proton adsorption as the kinetic bottleneck. As the pH increases to 7.0 and 9.0, HER activity decreases, as reflected in both the cathodic current densities and the EC-MS ion currents.

Under O_2_ atmosphere, the Sac/CoP system exhibits clear ORR activity. At alkaline pH (pH 9), the system demonstrates the highest ORR current density (−0.5 mA cm^–2^ at −0.8 V), with a positively shifted onset potential around −0.3 V. This behavior is consistent with the reduced overpotential and favorable ORR kinetics in alkaline environments. The Sac system shows negligible ORR activity across all pH values, emphasizing the essential catalytic role of CoP. At neutral (pH 7) and acidic (pH 5) conditions, the ORR current densities decrease to −0.4 mA cm^–2^ and −0.3 mA cm^–2^, respectively, at comparable potentials. The reduced ORR performance at lower pH can be attributed to higher overpotentials and slower reaction kinetics, as observed in the shifted onset potentials (−0.4 V at pH 7 and −0.6 V at pH 5). Despite this, the Sac/CoP system maintains consistent ORR activity across the pH range, further demonstrating its robustness and versatility.

Sac plays a significant role in stabilizing the CoP catalyst and facilitating the diffusion of gases and electrolytes. This is particularly evident in the chronoamperometric measurements, where stable current densities are observed across all potential steps, indicating minimal deactivation of the Sac/CoP system. Additionally, the Sac system serves as an effective control, clearly demonstrating that the catalytic activity for both HER and ORR is driven by the immobilized CoP. The combination of Sac and CoP creates a biomimetic environment that mimics the catalytic behavior of natural metalloenzymes, where diffusion-limited conditions govern substrate and product transport. The quiescent solution further reinforces this analogy, providing a diffusion-controlled regime that highlights the intrinsic catalytic properties of the system. To further investigate the role of diffusion in electrochemical performance, CVs were recorded at scan rates ranging from 10 mV s^–1^ to 550 mV s^–1^ (Figure S6a). The linearity observed in the *J* vs *v* ^1^/^2^ plot (Figure S6c) suggests a diffusion-controlled contribution,^45^ while the peak shifts with scan rate indicate electron transfer kinetics at the electrode interface^46^ (Figure S6b). These results confirm that the Sac system effectively modulates mass transport properties, stabilizing the catalytic process while allowing efficient charge transfer. The dual catalytic behavior of the Sac/CoP system for HER and ORR demonstrates its potential as a versatile mimetic electrocatalyst. HER performance is maximized under acidic conditions, where proton availability is highest, whereas ORR activity is optimized in alkaline environments, where reduced overpotentials favor oxygen reduction. This complementary behavior underscores the adaptability of the system across diverse electrochemical processes.

### Long-Term Stability Analysis

Figure 4d illustrates the variation in current as a function of time over 14 scans conducted at pH 9 (gray line), pH 7 (red line), and pH 5 (blue line) under N_2_-purged conditions with a scan rate of 10 mV s^–1^. The data provide a clear representation of the system’s electrochemical stability during repeated cycling, which is critical for evaluating the robustness of the Sac/CoP system in prolonged catalytic applications. Figure 4e summarizes these results by displaying the average current density at each pH, reinforcing the trend observed in Figure 4d. At pH 5, the highest cathodic current densities are observed, consistent with the system’s enhanced HER activity in an acidic environment. To further evaluate long-term stability, Figure 4f presents a CV recorded after 50 cycles, allowing a direct comparison with Figure 4d. While both figures illustrate the electrochemical response over multiple scans, Figure 4f provides additional insight into sustained performance over extended cycling. The stability of CoP is influenced by experimental parameters such as electrode surface interactions, pH value, and the robustness of the supporting Sac matrix. In this study, the immobilization of CoP within a Sac matrix on HEDGE significantly contributed to its electrochemical stability. The Sac layer not only provided mechanical support but also prevented significant catalyst lixiviation by maintaining the structural integrity of CoP during extended electrochemical cycling. To further investigate the origin of the current decay observed over the 50 cycles, FTIR analyses were performed on the Sac/CoP modified electrodes before and after electrochemical cycling. The spectra (Figure S9) show that the key vibrational bands associated with the corrin ring and the agarose matrix remain largely unchanged after cycling, with only a minor shift in the C–O stretching region. This subtle variation is attributed to local reorganization of the hydrogel network or weak interactions with the electrolyte, rather than any significant degradation of the composite. The preservation of molecular features confirms that the system retains its structural integrity, supporting the hypothesis that the observed current loss arises from mild surface effects or partial leaching, rather than from chemical decomposition of the catalytic material.

CV analysis revealed minimal changes in current density over various scans, demonstrating the system’s resilience in all pH values of these studies. At pH 5, where HER activity was most pronounced, the CoP exhibited stable cathodic current densities across 50 cycles with less than 22% decay over 50 cycles (Figure S8; Table S1), suggesting an effective electrochemical stability. At pH 7, a moderate current response is maintained throughout the scans, demonstrating the system’s capacity to operate in a neutral environment with consistent performance. Although the current density is lower than at pH 5, the lack of current decay indicates that the Sac/CoP composite retains its electrochemical activity and mechanical stability, even under less kinetically favorable conditions. At pH 9, the current response is the lowest, reflecting the reduced availability of protons for HER in alkaline conditions. However, the stability of the current over 14 cycles demonstrates that the system remains chemically and mechanically robust under these conditions. This is particularly important for applications that require operation in alkaline media, where long-term stability is often a challenge due to the potential degradation of catalytic materials. The consistent current profiles observed across all pH values highlight the Sac/CoP system’s excellent long-term stability, even under quiescent conditions. The Sac matrix maintains the structural integrity of the electrode by preventing catalyst lixiviation and ensuring efficient mass transport of reactants and products. The chronoamperometry experiment at −1.3 V vs Ag/AgCl for 11 h (Figure S5) assesses HER stability in the presence of O_2_. The 11-h electrolysis confirms the catalyst’s contribution to HER but also reveals a gradual current decrease, suggesting possible deactivation mechanisms like surface modification. Despite the presence of O_2_, HER activity remains stable over this period, reinforce the system’s durability. This behavior is commonly associated with surface restructuring and electrolyte species adsorption, which influence initial current fluctuations.^47^ The subsequent gradual decay can result from structural rearrangement of active sites, or the formation of passivating species on the electrode surface.^12,48^ Furthermore, the presence of O_2_ could contribute to oxidative deactivation or competitive adsorption effects, altering the catalyst local environment. Despite these challenges, the Sac matrix appears to mitigate severe deactivation by providing a controlled microenvironment that limits excessive leaching and enhances mass-transport properties, as observed in similar hydrogel-based catalysts.^49^

## Discussion

This study demonstrates the structural and functional integration of CoP into a Sac matrix supported on HEDGE, creating a biomimetic platform for dual electrocatalysis. The approach combines the catalytic properties of CoP with the stability and permeability of the agarose hydrogel, providing a system that mimics the localized and diffusion-limited environments typical of natural enzymatic processes.^50,51^ Spectroscopic analysis confirmed the retention of key functional groups from both the Sac and the CoP, while MEV-FEG micrographs revealed uniform coverage and the incorporation of CoP into the matrix. The EDS analysis further validated the structural integration, identifying the expected presence of cobalt along with supporting elements such as nitrogen and oxygen, which are critical for the CoP catalytic function.

The system exhibits catalytic activity for both HER and ORR, with distinct pH-dependent behaviors that align with its biomimetic design. Under acidic conditions (pH 5), HER activity was most pronounced, achieving cathodic current densities of approximately −1.2 mA cm^–2^ at −1.5 V. This is consistent with the high proton availability in acidic environments, which enhances reduction kinetics, like enzymatic systems that operate in proton-rich microenvironments.^52^ The Tafel slope of 266 mV dec^–1^, although higher than classical values for Volmer-type RDS suggests additional resistances possibly arising from local proton diffusion limitations or the influence of the Sac matrix on charge-transfer dynamics.^44^ This indicates that proton adsorption remains the dominant mechanism, drawing parallels with natural hydrogenase enzymes, which efficiently catalyze hydrogen evolution under comparable conditions.^53^ This behavior reflects the reduced overpotential and increased efficiency of CoP under alkaline conditions, comparable to the performance of metalloenzymes that catalyze oxygen reduction in basic cellular compartments. The agarose hydrogel plays an important role in mimicking the properties of biological environments, acting as a permeable matrix that facilitates selective diffusion of gases and electrolytes while stabilizing the catalytic CoP. This design replicates the natural architecture of metalloenzymes, where substrate access and product removal occur through localized diffusion pathways. Quiescent conditions further reinforce this analogy, as the absence of forced convection mimics the diffusion-limited regimes in which enzymes operate, emphasizing the intrinsic catalytic properties of the system.

## Conclusion

This study demonstrates the successful integration of CoP into an agarose hydrogel matrix supported on HEDGE, resulting in a biomimetic electrocatalytic system with dual functionality for the HER and ORR. The combination of morphological, spectroscopic, and electrochemical analyses supports the structural and functional stability of the composite, emphasizing its ability to replicate key features of natural enzymatic systems. The system exhibits distinct pH-dependent catalytic activity. This biomimetic system bridges the gap between biological principles and synthetic catalysis, offering insights into the design of artificial metalloenzymes and robust electrocatalysts. By mimicking the structural and functional properties of natural enzymes, the Sac/CoP composite demonstrates its potential for efficient and adaptable performance, emphasizing the importance of biomimetic strategies in advancing catalytic systems.

## Supplementary Material



## Notes

Details of material acquisition, micro-FTIR spectrum, EDS analysis, and additional electrochemical characterization: adsorption voltammograms of the material on GCE and HEDGE, chronoamperogram to assess stability, study of scan rate variation, and capacity fade of the material (PDF)
