# Supplementary material for: Biomimetic Cobalt Complex Stabilized by Hydrogel on High-Edge-Density Graphite for ORR and HER in Quiescent Solutions
Source: Langmuir. 2025 Aug 19;41(34):22738–48. doi: 10.1021/acs.langmuir.5c01683 (PMC12409881; doi:10.1021/acs.langmuir.5c01683)
Supplement: Supplementary file 1 [file la5c01683_si_001.pdf]

Supporting Information

Biomimetic Cobalt Complex Stabilized by Hydrogel on High-Edge-Density Graphite for ORR and HER in Quiescent Solutions

Fhysmélia F. Albuquerque[1], Rodrigo M. Iost[2], Gabriel C. Fonseca[1,3,5], Radhakrishnan Venkatkarthick [1], Jessica C. Pacheco[1], Rafael N. P. Colombo[1,4], Fabio H. B. Lima[1], Frank N. Crespilho[1]*

[1]São Carlos Institute of Chemistry, University of São Paulo (USP), 13560-970, São Carlos, Brazil.

[2]Department of Fundamental Chemistry, Institute of Chemistry, University of Sao Paulo, Av. Professor Lineu Prestes, 748-B4T, Butantã, Sao Paulo 05508-000, Brazil.

[3]Goiano Federal Institute of Education, Science and Technology, Campus Rio Verde, Rio Verde, GO 75901-970, Brazil.

[4] Chemistry Department, Federal University of São Carlos (UFSCar), 13565-905, São Carlos, SP, Brazil.

[5]BCMaterials, Basque Center for Materials, Applications and Nanostructures, UPV/EHU Science Park, Leioa 48940, Spain

* frankcrespilho@iqsc.usp.br

1. Chemicals 2
2. Micro-FTIR 2
3. EDS 3
4. Electrochemistry 4
5. RRDE Measurements 5
6. Current fade 6
7. Additional FTIR data 8

1. Chemicals

Glutaraldehyde (25%), cobalt porphyrin (CoP) (Figure S1), sodium hydroxide, and sulfuric acid (98%) were obtained from Sigma-Aldrich. Agarose and sodium sulfate were sourced from Kasvi and Êxodo, respectively. The graphite rod was acquired from Alpha Chemicals.

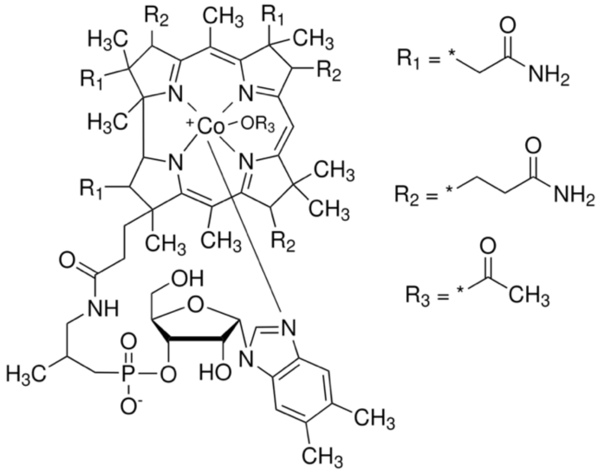

Figure S1. Chemical structure of hydroxocobalamin acetate (CoP), a cobalt corrin analogue of vitamin B12 employed as the active catalytic component.

2. Micro-FTIR

MicroFTIR analyses were performed using a Bruker Vertex 70V spectrometer coupled with a Hyperion 3000 microscope. Analyses were performed in reflectance mode and with a N_2-cooled mercury cadmium telluride (MCT) detector. By drop casting, solutions of the respective materials were placed on a gold plate and dried overnight. Spectra were recorded from the average of 128 accumulations, with a spectral resolution of 4 cm^{-1} and a spectral window of 620–4000 cm^{-1} using an objective lens with 36× magnification.

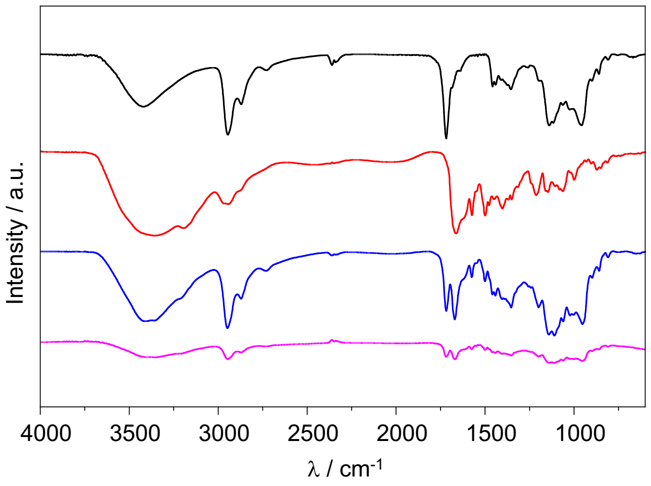

Figure S2. Micro-FTIR spectra recorded in the range of 620–4000 cm^{-1} to identify localized chemical interactions within Sac (black line), CoP (red line), Sac/CoP (blue line) and difference spectrum (CoP - Sac) (pink line).

3. EDS

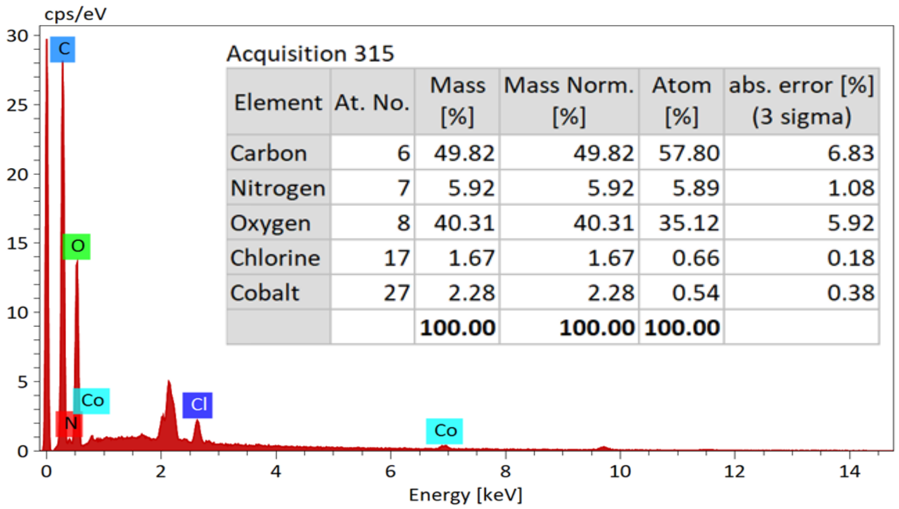

Element	At. No.	Mass [%]	Mass Norm. [%]	Atom [%]	abs. error [%] (3 sigma)
Carbon	6	49.82	49.82	57.80	6.83
Nitrogen	7	5.92	5.92	5.89	1.08
Oxygen	8	40.31	40.31	35.12	5.92
Chlorine	17	1.67	1.67	0.66	0.18
Cobalt	27	2.28	2.28	0.54	0.38
		**100.00**	**100.00**	**100.00**	

Figure S3. EDS data cobalt integration (2.28%) and the presence of key elements (C, N, O), verifying the successful modification of the hydrogel matrix. The acceleration voltage used for SEM imaging was 5.0 kV.

4. Electrochemistry

The performance of the HEDGE working electrode was compared to that of a conventional glassy carbon electrode (GCE) to assess its adsorption capacity and electron transfer efficiency, both crucial for studying the hydrogen evolution reaction (HER) and oxygen reduction reaction (ORR) of cobalt corrin complex (CoP). Consequently, the greater number of exposed edges in HEDGE, compared to GCE, markedly enhances its efficiency in electron transfer processes.

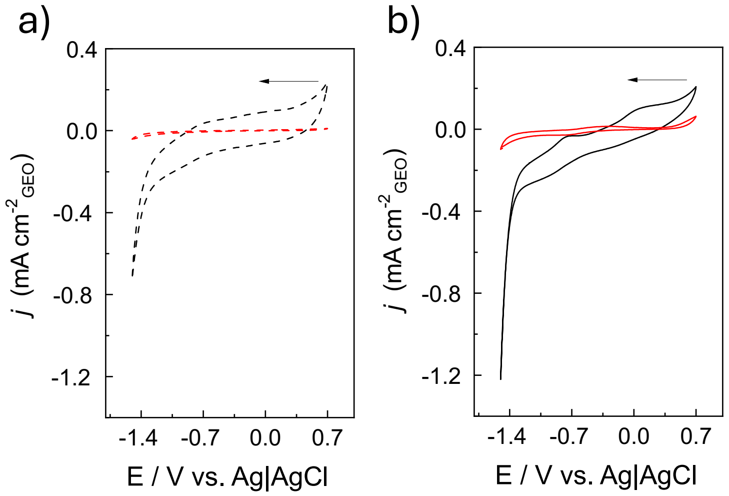

Figure S4. Cyclic voltammograms of **a)** HEDGE/Sac (black line) and GCE/Sac (red line) and **b)** HEDGE/Sac/CoP (black line) and GCE/Sac/CoP (red line) under N_2-saturated solutions at pH 5. Supporting electrolyte: Na_2SO_4 0.1 mol L^{-1}. Scan rate: 10 mV s^{-1}.

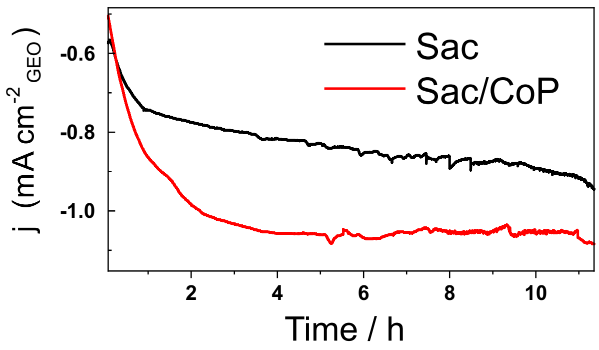

Figure S5. Chronoamperometry experiment conducted at -1.3 V vs. Ag/AgCl for 11 hours to evaluate HER stability in the presence of O_2.

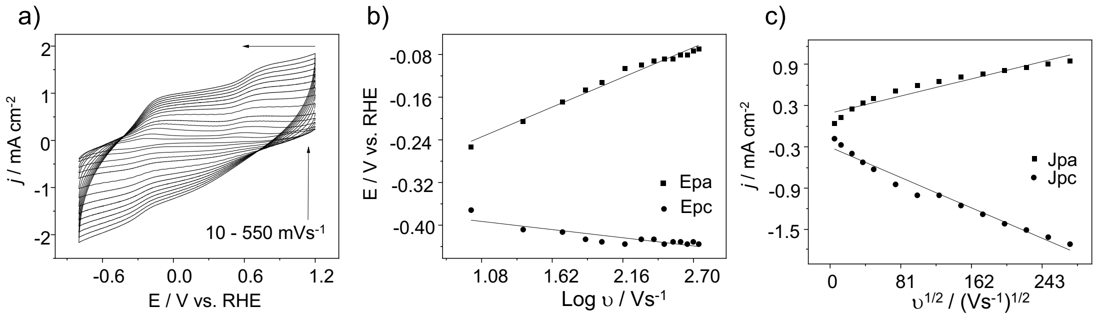

Figure S6. **a)** Cyclic voltammograms of HEDGE/Sac/CoP recorded at different scan rates, ranging from 10 to 550 mV s^{-1}; **b)** Laviron plot *E* (vs. Ag/AgCl) vs. log v and **c)** *J* vs. $v^{1/2}$ plot at pH 5. Supporting electrolyte: Na_2SO_4 0.1 mol L^{-1}.

5. RRDE Measurements

RRDE experiments were carried out using a gold disk–platinum ring electrode (Au/Pt RRDE), with an Ag/AgCl/Cl^-_{sat} electrode as reference and a platinum wire as counter electrode. Collection efficiency was tested before the experiments to ensure electrode performance. The Sac/CoP-modified gold disk was prepared by drop-casting 50 µL of the catalyst suspension, followed by drying under vacuum at room temperature. Measurements were performed in 0.1 M Na_2SO_4 (pH 9) under saturated dissolved O_2, N_2, and N_2 with added H_2O_2, at a rotation speed of 1600 rpm. The disk potential was scanned at 10 mV s^{-1}, while the ring potential was held constant at +0.3 V vs. Ag/AgCl. The appearance of a ring current under O_2-saturated conditions, which is absent under N_2-purged conditions, is attributed to the ORR at the disk.

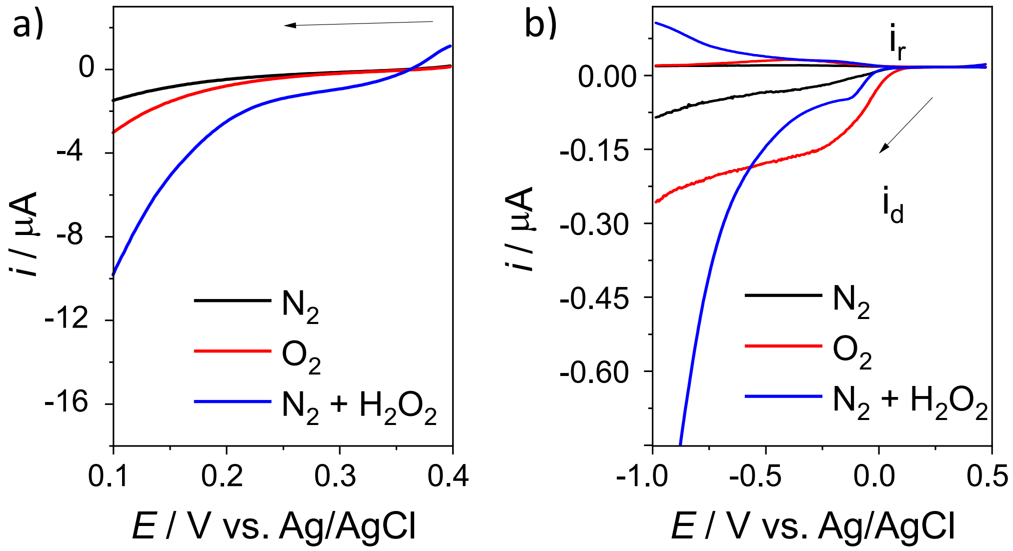

Figure S7. a) LSV of the Pt ring electrode in solutions saturated with N_2 (black line), O_2 (red line), and N_2 + H_2O_2 5 mM (blue line); **b)** LSV of Au disk electrode and the corresponding ring current at 0.3 V in solutions saturated with N_2 (black line), O_2 (red line), and N_2 + H_2O_2 5 mM (blue line).

6. Current fade

The current fade was calculated using the initial current from third cycle (j_{pa} = 0.36 mA cm^{-2}) as the reference. The percentage loss at each cycle was determined using the equation S1.

$$\text{Fade (\%)} = \frac{j_{\text{initial}} - j_{\text{current cycle}}}{j_{\text{initial}}} \times 100 \qquad \text{(S1)}$$

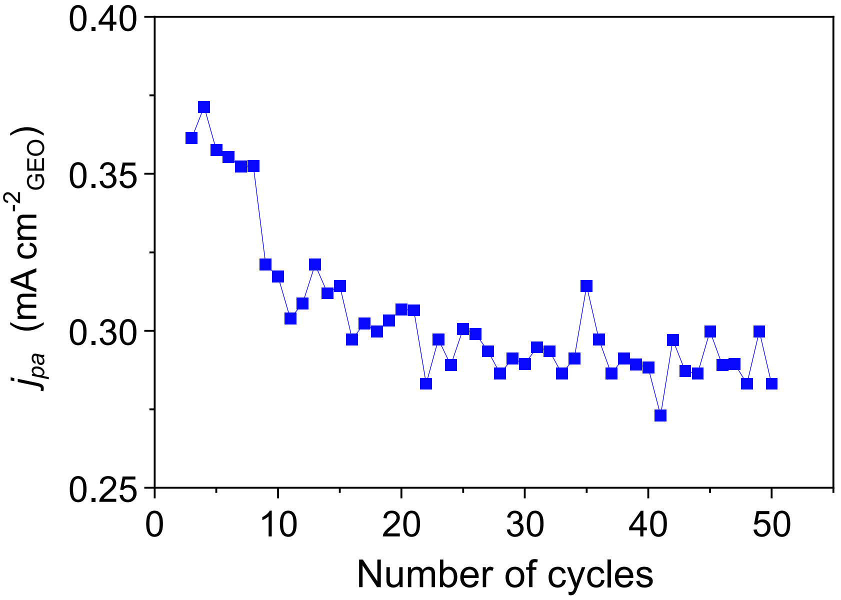

Figure S8. Evolution of the peak current density (j_{pa}) over multiple cycles, demonstrating the capacity fade of HEDGE/Sac/CoP. Supporting electrolyte: Na_2SO_4 0.1 mol L^{-1}. Scan rate: 10 mV s^{-1}.

The results are summarized in **Table S1**, which shows the current density (j_{pa}) and the corresponding fading percentage across selected cycles. As shown, a progressive reduction in current is observed over time.

Table S1. Current fade (%) with respect to the cycle number.

Cycle	j_{pa} (mA cm^{-2}$_{GEO}$)	Fade (%)
**3**	0.36	0.00
**11**	0.30	15.91
**21**	0.31	15.15
**31**	0.29	18.45
**41**	0.27	24.44
**50**	0.28	21.66

The results demonstrate a low current loss over the evaluated cycles, highlighting the stability of the system. At the 50st cycle, the current decreased from 0.36 mA cm^{-2} to 0.28 mA cm^{-2}, representing a total loss of only 0.08 mA cm^{-2}. In percentage terms, the fade reached 21.7%, which, given the number of cycles tested, indicates a gradual and controlled degradation. The low current loss observed is a positive result, suggesting that the system maintains its electrochemical activity over a significant number of cycles.

7. Additional FTIR data

To assess possible structural changes after electrochemical cycling, FTIR spectra were recorded before and after 50 voltammetric cycles. The results are shown in **Figure S9**.

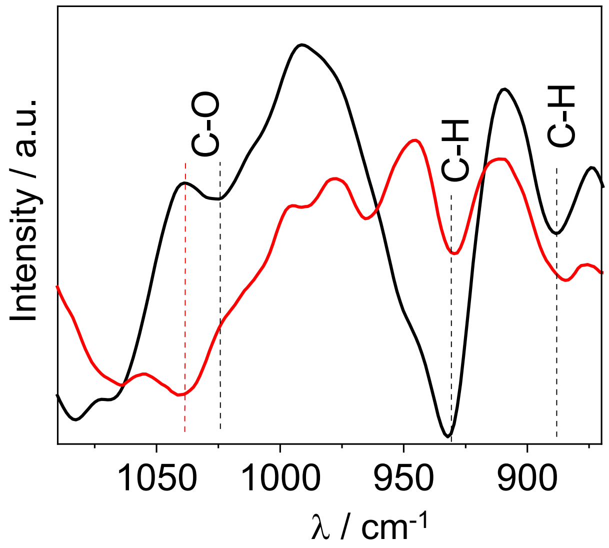

Figure S9. FTIR spectra recorded before (black line) and after (red line) 50 voltammetric scans.
